# Extra-haematological manifestations related to human parvovirus B19 infection: retrospective study in 25 adults

**DOI:** 10.1186/s12879-018-3227-1

**Published:** 2018-07-04

**Authors:** Marion Dollat, Benjamin Chaigne, Grégoire Cormier, Nathalie Costedoat-Chalumeau, François Lifermann, Alban Deroux, Emilie Berthoux, Emmanuelle Dernis, Thomas Sené, Gilles Blaison, Olivier Lambotte, Benjamin Terrier, Jérémie Sellam, Luc De Saint-Martin, Laurent Chiche, Nicolas Dupin, Luc Mouthon

**Affiliations:** 1 Service de Médecine Interne, Centre de Référence Maladies Systémiques Autoimmunes Rares d’Ile de France, Hôpital Cochin, Assistance Publique-Hôpitaux de Paris (AP-HP), Université Paris Descartes, Paris, France; 2 Service de Rhumatologie, Centre Hospitalier Départemental Vendée, La Roche-sur-Yon, France; 3 Service de Médecine Interne, Centre Hospitalier de Dax - Côte d’Argent, Dax, France; 4 Service de Médecine Interne, Centre Hospitalier Universitaire Grenoble Alpes, Université Grenoble Alpes, Grenoble, France; 5 Service de Médecine Interne, Centre Hospitalier Saint-Joseph Saint-Luc, Lyon, France; 6 Service de Rhumatologie, Centre Hospitalier – Le Mans, Le Mans, France; 7 Service de Médecine Interne, Hôpital Foch, Suresnes, France; 8 Service de Médecine Interne, Centre Hospitalier Louis Pasteur, Colmar, France; 9 Service de Médecine Interne et Immunologie, Hôpital Bicêtre, AP-HP, Université Paris-Sud, Le Kremlin-Bicêtre, France; 10 Service de Rhumatologie, Hôpital Saint-Antoine, AP-HP, Université Pierre et Marie Curie, Paris, France; 11 Service de Médecine Interne, Centre de Référence Maladies Systémiques Autoimmunes Rares Nord et Ouest, Centre Hospitalier Régional et Universitaire de Brest, Université de Bretagne Occidentale, Brest, France; 12 Service de Médecine Interne, Hôpital Européen, Marseille, France; 13 Service de Dermatologie, Hôpital Cochin, AP-HP, Université Paris Descartes, Paris, France; 14 Service de médecine interne, Hôpital Cochin, 27, rue du faubourg Saint-Jacques, 75679 Paris Cedex 14, France

**Keywords:** Human parvovirus B19, Arthritis, Vasculitis, Glomerulonephritis, Peripheral neuropathy, Intravenous immunoglobulin, lupus

## Abstract

**Background:**

To describe extra-haematological manifestations associated with human parvovirus B19 (HPV-B19) infection.

**Methods:**

We conducted a nationwide multicentre study to retrospectively describe the characteristics and outcome of extra-haematological manifestations in French adults.

**Results:**

Data from 25 patients followed from 2001 to 2016 were analysed. Median age was 37.9 years (range: 22.7–83.4), with a female predominance (sex ratio: 4/1). Only 3 patients had an underlying predisposing condition (hemoglobinopathy or pregnancy). The most common manifestations were joint (80%) and skin (60%) involvement. Four patients (16%) had renal involvement (endocapillary proliferative or membranoproliferative glomerulonephritis, focal segmental glomerulosclerosis). Three patients (12%) had peripheral nervous system involvement (mononeuritis, mononeuritis multiplex, Guillain-Barré syndrome) and 2 (8%) presented muscle involvement. Other manifestations included hemophagocytic lymphohistiocytosis (*n* = 1), myopericarditis and pleural effusion (*n* = 1), and lymphadenopathy and splenomegaly mimicking lymphoma with spleen infarcts (*n* = 1). Immunological abnormalities were frequent (56.5%). At 6 months, all patients were alive, and 54.2% were in complete remission. In 2 patients, joint involvement evolved into rheumatoid arthritis. Six patients (24%) received intravenous immunoglobulin (IVIg), with a good response in the 3 patients with peripheral nervous system involvement.

**Conclusions:**

HPV-B19 infection should be considered in a wide range of clinical manifestations. Although the prognosis is good, IVIg therapy should be discussed in patients with peripheral nerve involvement. However, its efficacy should be further investigated in prospective studies.

## Background

Human parvovirus B19 (HPV-B19) is a small DNA virus discovered in 1974 in the serum of healthy blood donors [1]. Primary infection is often asymptomatic [2]. Its pathogenic role was first reported in patients with sickle cell disease who developed aplastic crisis. It was then recognized as a causative agent of erythema infectiosum, hydrops fetalis and fetal deaths, and pure red cell aplasia (PRCA) in immunocompromised patients [3]. In young adults, HPV-B19 infection is also associated with joint manifestations [2, 4–10].

HPV-B19–related extra-haematological manifestations have been described, including glomerulonephritis, myositis, myocarditis, vasculitis and nervous system involvement, [11–17]. Furthermore, HPV-B19 infection can favour the development of auto-immune manifestations [18]. Limited data have been reported on the outcome and management of extra-haematological manifestations of HPV-B19 infection.

Intravenous immunoglobulin (IVIg) preparations have been proposed to treat persisting infection in immunocompromised patients [19]. Although their efficacy is established for treating HPV-B19 PRCA, data on IVIg treatment for extra-hematological manifestations are scarce and rely on case reports and small series [20–23]. In the present work, we aimed to better characterize the spectrum of extra-haematological manifestations related to HPV-B19 infection in adult patients, with a particular focus on treatment and outcome.

## Methods

### Patients

We conducted a nationwide multicenter study involving members of the Société Nationale Française de Médecine Interne (SNFMI) and the Club Rhumatismes et Inflammations (CRI). Practitioners who had cared for patients with extra-hematological manifestations attributed to HPV-B19 infection between January 2001 and March 2016 were invited to anonymously report cases. The 1500 members of the SNMFI were contacted through the SNFMI website (http://www.snfmi.org) and mailing list and the 2500 members of the CRI were contacted through the CRI website (http://www.cri-net.com) and mailing list. Patients over 18 years old with at least one extra-haematological manifestation associated with HPV-B19 infection were included. HPV-B19 infection diagnosis was based on positive anti-HPV-B19 IgM serology and/or detection of HPV-B19 DNA by polymerase chain reaction (PCR) in peripheral blood and/or bone marrow or another tissue. PCR amplification could be quantitative, semi-quantitative, or qualitative. Patients with isolated hematological manifestations were excluded.

### Patient data

A standardized form was used to collect data on age; sex; underlying conditions; HPV-B19 infection diagnosis and manifestations; laboratory data: C-reactive protein (CRP) level, creatininemia, serum transaminase level, creatine phosphokinase level, antinuclear antibodies (Abs) (ANA), anti-double stranded DNA (anti-dsDNA) Abs, anti-extractable nuclear antigen (anti-ENA) Abs, lupus anticoagulant; anti-phospholipid Abs, anticardiolipin Abs, complement proteins C3 and C4, haemolytic complement activity, rheumatoid factor, anti-citrullinated Abs (ACPA), anti-neutrophil cytoplasmic Abs (ANCA), and cryoglobulinemia; treatment; and outcome. Day 1 was considered the day of the first clinical symptom. Outcomes were determined at 1, 6, and 12 months, when available.

### Ethics, consent and permissions

This study was conducted in compliance with the protocol of Good Clinical Practices and Declaration of Helsinki principles. In accordance with French law (Loi de Santé publique 2004 - Loi n° 2004–806 du 9 août 2004), the need for consent was deemed unnecessary and formal approval from an ethical committee was not required for this study.

## Results

In total, 15 practitioners answered our call for clinical cases and 25 patients with extra-haematological manifestations related to HPV-B19 infection were included. Their characteristics are in Table 1. Eighteen (72%) were younger than 40 years. The female-to-male ratio was 4/1. Only 4 patients had a significant underlying condition: 2 had hemoglobinopathy (patient 7: alpha-thalassemia, patient 17: sickle cell disease), one had a history of cutaneous lupus (patient 12), and one was pregnant (23 weeks, patient 4). Median time to diagnosis was 13 days (range: 0–197). In 7 patients (28%), the occurrence of extra-haematological manifestations was preceded by an influenza-like illness. Three patients (12%) had a contact with a child with erythema infectiosum. Inflammatory syndrome, when present (19/24 cases, 79%), was moderate (median CRP level: 19 mg/L, range: 0–132). The diagnosis of HPV-B19 infection was based on positive serology (IgM) in 17 patients (68%), positive blood PCR in 2 (8%), or both in 6 (24%). In one patient, a bone-marrow biopsy was performed; PCR findings were positive for HPV-B19 DNA.

**Table 1:** Clinical and biological characteristics, treatment and outcome in 25 patients with extra-haematological manifestations associated with human parvovirus B19 (HPV-B19) infection

N°	Age/ Sex	Type of damage	HPV-B19 markers		CRP, mg/l	Immunological abnormalities	Treatment	Clinical status during follow-up			Residuals symptoms^a^	PCR negativation^a^/ Time to negativation, months	Follow-up, months
			IgM/IgG	Blood/TissueDNA				M1	M6	M12			
1	40/F	Symmetrical polyarthralgia, palpable purpura with subcutaneous nodules	+/+	NP/NP	58	None	None	PR	CR	CR	None	NA/NA	12
2	28/F	Symmetrical polyarthralgia, myalgia, exanthema	+/+	NP/NP	4	None	NSAIDs	PR	CR	CR	None	NA/NA	12
3	24/M	Symmetrical polyarthralgia; exanthema, PPGSS	+/+	NP/NP	< 3	None	CS 1 mg/kg/d	CR	CR	CR	None	NA/NA	12
4	28/M	Symmetrical polyarthralgia	+/+	NP/NP	9	ANA+	None	CR	CR	CR	None	NA/NA	12
5	49/M	Arthromyalgia with axial involvement, elevated liver enzymes	+/+	NP/NP	23	NP	NSAIDs	CR	CR	CR	None	NA/NA	12
6	37/F	Polyarthritis; slapped-cheek rash, hands edema	+/+	NP/NP	21	ANA+, LA+	None	PR	CR	CR	None	NA/NA	12
7	38/F	HLH, acute myopericarditis, pleural effusion; polyarthritis	+/+	+/+(BM)	4	ANA+, RF+, ACPA+, aCL Abs+ (IgG), anti β2GP1 Abs+ (IgG)	NSAIDs, IVIg, CS 10 mg/d, MTX, anti- TNF-α, anti-IL6R	S	S	S	IPPJ synovitis^b^	Yes/11	15
8	38/M	Symmetrical polyarthralgia; thighs myalgia	+/+	NP/NP	35	ANA+, anti-dsDNA+, aCL Abs+ (IgG, IgM), C4↓	NSAIDs, HCQ, CS 30 mg/d, MTX	S	PR	PR	Arthralgia	NA/NA	12
9	24/F	Febrile acute polyarthritis with axial involvement	+/+	NP/NP	132	ANA+	NSAIDs	PR	PR	PR	Migratory polyarthragia, asthenia	NA/NA	13
10	38/F	Distal polyarthritis	+/+	+/NP	< 3	ANA+, anti-dsDNA+, C4↓	NSAIDs	PR	PR	–	None	No/NA	9
11	34/F	Polyarthritis; exanthema; asthenia	+/+	NP/NP	114	ANA+, RF+, ANCA+	NSAIDs, CS 0.1 mg/kg/d, MTX, HCQ + NSAIDs	W	S	S	None^b^	NA/NA	89
12	32/F	Polyarthritis; exanthema, livedo; hands and feet edema; transient proteinuria	+/+	+/NP	32	None	NSAIDs, colchicine, IVIg, HCQ, myorelaxant, antidepressants	S	S	S	Asthenia, arthromyalgia	Yes/21	48
13	42/F	Polyarthritis, asthenia	+/+	NP/NP	NA	ANA+, anti-dsDNA+, aCL Abs+ (IgG, IgM)	NSAIDs	PR	CR	–	None	NA/NA	6
14	39/F	Cyclical and diffuse arthromyalgia, exanthema	+/+	+/NP	12	None	Antidepressants CS 1 mg/kg	S	S	S	None	Yes/18	25
15	24/F	Polyarthritis, exanthema	+/+	NP/NP	7	ANA+, RF+, ACPA+	NSAIDs, CS 1 mg/kg	PR	CR	CR	None	NA/NA	12
16^c^	37/F	Symmetrical and febrile polyarthralgia, calves myalgia	+/+	+/NP	34	RF+, C4↓, abundant mixed cryoglobulin (type II)^d^	NSAIDs	PR	CR	–	None	No/NA	6
17	44/M	Nephrotic syndrome, ARF	NA/NA	+/NP	60	NA	Pulsed MP, oral CS 1 mg/kg/d, ACEIs + ARBs, IVIg	W	S	W	Nephrotic syndrome, ESRD	No/NA	23
18	56/F	Febrile polyarthritis; palpable purpura; nephritic syndrome; ARF; asthenia	+/NA	+/NP	31	ANA+, anti-dsDNA+, ANCA+, C4↓	Diuretics, ACEIs	PR	CR	CR	None	NA/NA	30
19	22/F	Acute nephritic syndrome	+/+	NP/NP	17	Anti-dsDNA+, ANCA+, C4↓	Diuretics	PR	CR	CR	None	NA/NA	12
20	37/F	Pure sensory axonal mononeuritis multiplex	−/+	+/NP	< 3	None	CS 1 mg/kg/d, AEDs, IVIg	W	W	PR	Hypoesthesia	No/NA	25
21	42/F	Left ulnar mononeuritis, necrotic purpura with PPGSS	+/+	+/NP	8	ANA+, RF+, cryoglobulin (type III)	CS 20 mg/d, AEDs, IVIg	W	PR	–	Numbness 5th finger	No/NA	7
22	30/F	GBS, polyarthralgia, exanthema, lymphadenopathy	+/−	+/− (CSF)	40	None	IVIg	PR	CR	CR	None	No but ↓↓/NA	18
23	37/F	PPGSS, palpable purpura; symmetrical polyarthralgia	+/+	+/NP	7	None	NSAIDs, CS 1 mg/kg/d	CR	–	–	None	No/NA	4
24	39/M	Palpable purpura & subcutaneous nodules; arthralgia	+/+	−/− (skin)	12	Very low mixed cryoglobulin (type II)	NSAIDs, CS 1 mg/kg/d, colchicine, dapsone	PR	PR	CR	None	NA/NA	13
25	83/F	Asthenia, lymphadenopathy, splenomegaly with spleen infarcts, PPGSS	+/+	-/NP	40	ANA+	None	PR	CR	CR	None	NA/NA	14

^a^At the end of follow-up

^b^Evolution towards rheumatoid arthritis, in remission in patient N°11

^c^This case has already been published separately [45]

^d^HPV-B19 DNA was more abundant in the cryoprecipitate than in the serum sample

Abbreviations: *Abs* antibodies, *ACEIs* angiotensin-converting enzyme inhibitors, *aCL* anticardiolipin, *ACPA* anticitrullinated antibodies, *AEDs* antiepileptic drugs, *ANA* antinuclear antibodies, *ANCA* anti-neutrophil cytoplasmic antibodies; anti-dsDNA, anti-double stranded DNA antibodies; *anti-IL6R* anti-interleukin-6 receptor, *anti-TNF-α* anti-tumor necrosis factor α, *ARBs*, angiotensin II receptor antagonists, *ARF* acute renal failure, *BM* bone marrow, *C4* complement factor 4, *CP* complete remission, *CS* corticosteroids, *CSF* cerebrospinal fluid, *d* days, *w* weeks, *ESRD* end stage renal disease, *GBS* Guillain-Barré syndrome, *HCQ* hydroxychloroquine, *HLH* hemophagocytic lymphohistiocytosis, *IVIg* intravenous immunoglobulin, *LA* lupus anticoagulant, *MP* methylprednisolone, *MTX* methotrexate, *NA* not available, *NP* not performed, *NSAIDs* nonsteroidal anti-inflammatory drug, *PPGSS* papular-purpuric gloves and socks syndrome, *PR* partial remission, *RF* rheumatoid factor, *S* stable, *W* worsening

### Clinical manifestations

The most common extra-haematological manifestations were joint and skin involvement. Common manifestations were also kidney and PNS involvement (Table 2). Less common manifestations included myopericarditis, muscle involvement and vasculitis.

**Table 2:** Clinical characteristics in 25 patients with extra-haematological manifestations associated with human parvovirus B19 infection

Age, years, median (range)	37.9 (22.7–83.4)
Female sex	20 (80)
Joint involvement	20 (80)
Symmetrical polyarthralgia	11 (44)
Arthritis	9 (36)
Skin involvement	15 (60)
Exanthema	8 (32)
Palpable purpura	5 (20)
PPGSS	4 (16)
Periflexural pattern	2 (8)
Other	4 (16)
Renal involvement	4 (16)
Endocapillary proliferative GN	1 (4)
Membranoproliferative GN + FSGS	1 (4)
Undetermined	2 (8)
Neurological involvement	3 (12)
Mononeuritis	1 (4)
Mononeuritis multiplex	1 (4)
Guillain-Barré syndrome	1 (4)
Other	11 (44)
Myalgia	5 (20)
Asthenia	3 (12)
Elevated liver enzymes	1 (4)
Myopericarditis and pleural effusion	1 (4)
Spleen infarcts	1 (4)

Data are n (%) unless indicated

Abbreviations: *FSGS* focal segmental glomerulosclerosis, *GN* glomerulonephritis, *PPGSS* papular-purpuric gloves and socks syndrome

#### Joint involvement

Twenty patients had joint manifestations; 11 (44%) presented symmetrical polyarthralgia involving peripheral joints. Nine (36%) patients had synovitis; 3 (12%) had acute febrile polyarthritis. Wrists (*n* = 14, 56%), ankles (*n* = 14, 56%), knees (*n* = 14, 56%) and hands (*n* = 11, 44%), including proximal and distal inter-phalangeal joints, were more often involved, whereas elbows (*n* = 7, 28%), shoulders (*n* = 6, 24%), feet (*n* = 2, 8%) and hips (*n* = 1, 4%) concerned less patients. Axial involvement was identified in 2 patients (8%).

#### Skin involvement

**Fig. 1:**
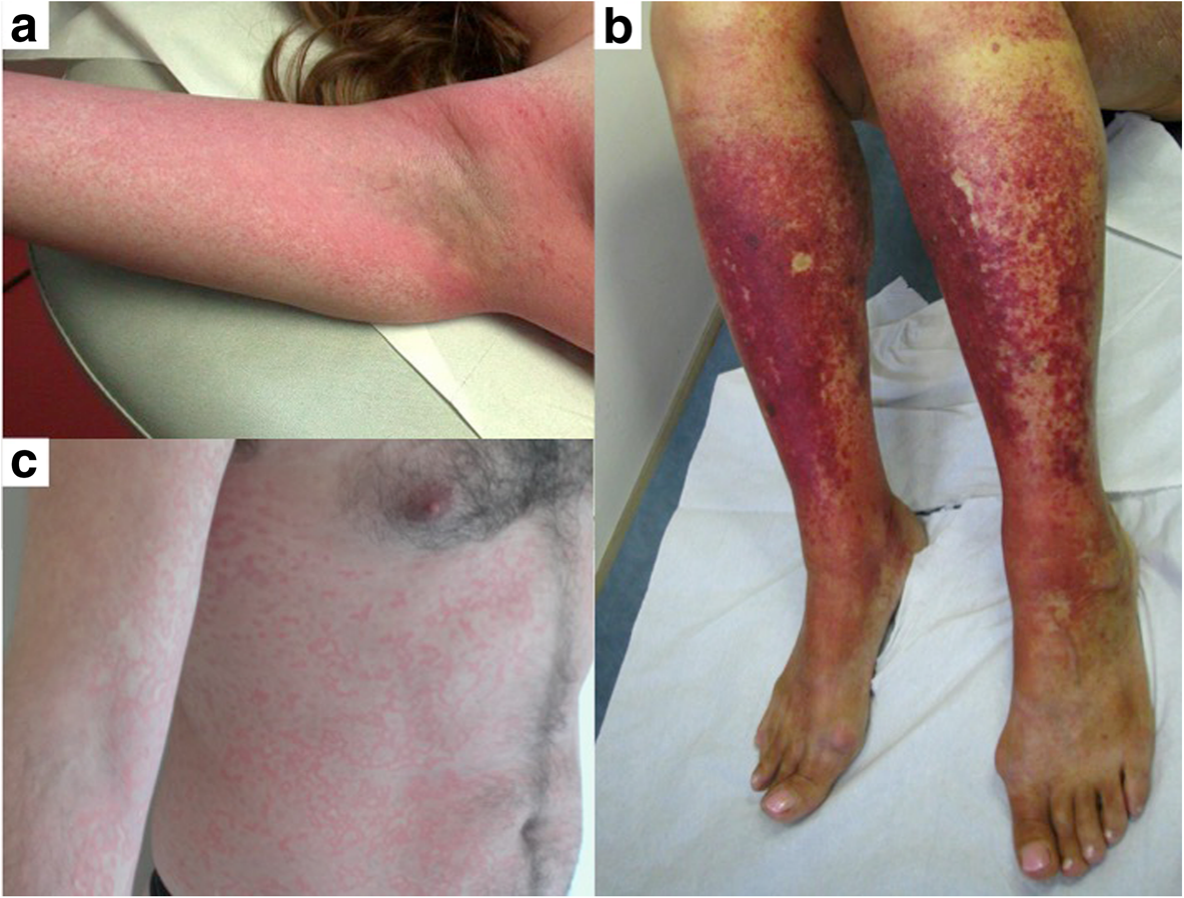
Skin manifestations related to human parvovirus B19 infection. Panel **a**: periflexural purpuric exanthema. Panel **b**: purpuric lesions of the lower limbs in a patient with the gloves and socks pattern. Pnael **c**: reticulate and annular exanthema of the trunk

Fifteen patients had skin manifestations. Skin lesions included erythema (*n* = 9, 36%), palpable purpura (*n* = 5, 20%), papules (*n* = 2, 8%), and nodules (*n* = 2, 8%), and were frequently associated. Five (20%) patients reported pruritus. Exanthema affected legs (*n* = 4, 16%), trunk (*n* = 3, 12%), arms (*n* = 2, 8%), and was generalized in one patient (Fig. 1). The face was involved in one woman, with the “slapped cheek” sign. Four patients presented with papulo-purpuric gloves-and-socks syndrome (PPGSS) (Fig. 1). A periflexural involvement was reported in 2 patients (8%) (Fig. 1), with petechial purpura in a baboon syndrome-like distribution in one. Subcutaneous nodules were identified in 2 patients (8%), localized on thighs or feet and forearms. Oedema was detected in 3 patients (12%), involving hands, feet, or both. One patient presented livedo localized to the lower limbs. Skin biopsies performed in 4 patients (16%) revealed leukocytoclastic vasculitis in 2, fibrinoid necrosis and capillary thrombosis with immunoglobulins and complement deposits in one and Sweet’s syndrome in one.

#### Renal involvement

Four patients had renal involvement. Transient moderate proteinuria was found in one patient. Two patients presented generalized oedema and acute nephritic syndrome and one had nephrotic syndrome with acute renal failure. Kidney biopsy was performed in 2 patients, revealing a focal and segmental endocapillary proliferative glomerulonephritis (GN) with C3 and kappa light chain deposits in the first patient and membranoproliferative GN with focal segmental glomerulosclerosis lesions in the other. When performed (*n* = 1), immunohistochemical study was negative for HPV-B19.

#### Neurological involvement

Three patients showed neurological involvement. One patient presented mononeuritis multiplex with progressive sensory disorders. Electrophysiological examinations revealed a distal and pure sensory involvement predominantly affecting small fibres. One patient complained of painful paresthesia and dysesthesia in the left ulnar nerve territory, with electrophysiological study suggesting an isolated ulnar mononeuritis with sensory-motor disorders. In one patient, clinical and electrophysiological examination led to the diagnosis of Guillain-Barré syndrome, restricted to lower limbs. In this patient, PCR findings for HPV-B19 DNA in cerebrospinal fluid were negative.

#### Other

Two patients had lower-limb myalgia with inflammatory muscular infiltration revealed by MRI but normal serum creatine phosphokinase level. One patient had abundant mixed cryoglobulin. Three patients (12%) had transient and moderate cytolytic hepatitis. One patient initially presented an acute severe clinical picture combining hemophagocytic lymphohistiocytosis, pleural effusion, and myopericarditis. One patient had febrile lymphadenopathy and splenomegaly mimicking lymphoma with multiple spleen infarcts: inguinal lymph node biopsy showed adenitis. Four other patients also showed lymphadenopathy.

Of note, extra-haematological manifestations were associated with haematological biological disorders in 11 patients (44%) and included anemia (*n* = 8, 32%), lymphocytopenia (*n* = 4, 16%), thrombocytopenia (*n* = 2, 8%), and pancytopenia (*n* = 1, 4%). Anemia was non-regenerative and moderate in 6 patients. In these patients, median [interquartile range] reticulocyte count was 65,000 [36000–167,500] cell/mm^3^. Among the 3 patients who underwent bone-marrow aspiration, none had PRCA.

### Immunological abnormalities

Immunological tests were available for 23 (92%) patients, with abnormalities detected in 13 (56.5%). ANA were present in 9 patients (39.1%; median [interquartile range] titre 80 [80–160]), with a speckled fluorescence; 5 (21.7%) had anti-dsDNA Abs (median [interquartile range] titre 99 [48–187]). None had anti-ENA Abs. Low C4 fraction level was observed in 7 of 18 patients (38.9%) (median [interquartile range] level 0.12 [0.095–0.14] g/L) including 4 with anti-dsDNA Abs. C3 fraction level was available and normal in 17 patients (100%). Anti-phospholipid Abs were found in 4 of 11 patients (36.4%): anti-cardiolipin Abs (IgG, *n* = 3; IgM, n = 2), anti-β2-glycoprotein1 Abs (IgG, *n* = 1), or lupus anticoagulant (*n* = 1). Six of 17 patients (35.3%) had rheumatoid factor, including 2 with ACPA. Three of 7 patients (42.9%) had mixed cryoglobulinemia: type II (2 cases), or type III (*n* = 1). ANCA were present in 3 of 15 patients (20%). In 5 of the 6 patients with repeated immunological tests, autoantibodies disappeared after 1 or 2 months. One patient had persistent anti-dsDNA Abs on ELISA 5 months after onset.

### Treatment and outcome

After a median (range) follow-up of 12 (4–89) months, every patient was alive. Four patients (16%) were in complete remission at 1 month, 13/24 (54.2%) at 6 months, and 12/20 (48%) at 12 months (Table 3). Four patients (16%) showed improvement without any treatment. Non-steroidal anti-inflammatory drugs (NSAIDs) and corticosteroids were the most frequently prescribed treatments, whereas 6 patients received IVIg (Table 3). Out of the 11 patients who received corticosteroids, 5 patients received corticosteroids before the diagnosis of HPV-B19 infection. In those 5 cases, corticosteroids were mostly empirically chosen due to the presence of joint involvement. Regarding the 6 other patients, corticosteroids were prescribed because of skin, neurological or kidney involvement. Remission or stabilisation was noted at 12 months in every patient who received corticosteroids except for one patient with nephrotic syndrome. Complete remission was noted in three patients.

**Table 3:** Treatment and outcome in patients with extra-haematological manifestations associated with HPV-B19 infection (*n* = 25)

Treatment (*n* = 25)	
None	4 (16)
NSAIDs	12 (48)
Corticosteroids	11 (44)
IVIg	6 (24)
Outcome	
At 6 months (*n* = 24)	
Complete remission	13 (54.2)
Partial remission	5 (20.8)
At 12 months (*n* = 20)	
Complete remission	12 (60)
Partial remission	3 (15)

Data are n (%) unless indicated

Abbreviations: *IVIg* intravenous immunoglobulin, *NSAIDs* non-steroidal anti-inflammatory drugs

Thirteen patients (52%) with joint involvement received NSAIDs: complete remission was observed with this treatment alone in 6 patients. Corticosteroids were prescribed to 5 patients (20%): 2 achieved complete response and one partial response. Two patients with joint involvement received IVIg, without efficacy (Table 4). Six patients showed persistent arthralgia or arthritis after 1 year of follow-up, notably 2 in whom rheumatoid arthritis (RA) developed.

**Table 4:** Treatment regimen and outcome for 6 patients who received intravenous immunoglobulin for extra-haematological manifestations associated with HPV-B19 infection

Predominant involvement	Patient 7	Patient 12	Patient 17	Patient 20	Patient 21	Patient 22
	Chronic polyarthritis	Chronic poly-arthralgia	Membrano-proliferative GN + FSGS	Mononeuritis multiplex	Mononeuritis	Guillain-Barré syndrome
Treatment regimen						
IVIg courses, n	1	1	20	20	1	1
IVIg first dose, g/kg	2	2	2	2	2	2
IVIg total dose, g/kg	2	2	36	37	2	2
Additional treatment	NSAIDs	NSAIDs	CS, ACEIs, ARBs	CS, AEDs	None	None
Outcome						
Clinical response M1	Stable	Stable	Stable	Worse	Stable	Partial
Clinical response M2	Stable	Stable	Stable	Partial	Stable	Partial
Clinical response M6	Stable	Stable	Stable	Partial	Partial	Complete
HPV-B19 PCR negativity	Yes	Yes	No	No	No	No
Time to PCR negativity, months after diagnosis/1st course	11/06	21/12	–	–	–	–

Abbreviations: *ACEIs* angiotensin-converting enzyme inhibitors, *AEDs* antiepileptic drugs, *ARBs* angiotensin II receptor antagonists, *CS* corticosteroids, *FSGS* focal segmental glomerulosclerosis, *GN* glomerulonephritis, *IVIg* intravenous immunoglobulin, *M* month, *NSAIDs* non-steroidal anti-inflammatory drugs, *PCR* polymerase chain reaction

Exanthema resolved spontaneously in all patients in 24 to 72 h. Corticosteroids were used in every patient with cutaneous vasculitis or Sweet’s syndrome and led to complete resolution in a few days or weeks except for one patient who required 9 months of treatment (patient 24).

The 2 patients with mononeuritis received corticosteroids with IVIg. IVIg was prescribed 15 days after corticosteroids in one and after 5 months in the other. Both patients showed mild neurological sequelae (Table 4). Guillain-Barré syndrome symptoms completely resolved after a single course of IVIg.

Finally, regarding renal involvement, end-stage renal disease (ESRD) developed in one patient (patient 17) despite monthly courses of IVIg. The patient with transient proteinuria did not show relapse and the 2 others recovered in 3 months.

PCR for HPV-B19 DNA was repeatedly performed during follow-up in 10 patients: 3 were negative for viremia, at 11 and 21 months in 2 patients who had received IVIg, and at 18 months in the third patient, who had only received corticosteroids.

## Discussion

HPV-B19 is a common pathogen in humans and mainly affects children and young adults. Seroprevalence in developed countries is 40 to 60% in adults older than 20 years and reaches over 90% in older people [24]. In adults, primary infection is asymptomatic or follows a two-phase benign course: an early phase with fever and nonspecific influenza-like symptoms, and a secondary phase with erythema, arthralgia, and appearance of anti-HPV-B19 IgM [2, 25, 26]. Haematological disorders associated with HPV-B19 are well identified and commonly described [27, 28]. Conversely, extra-haematological manifestations have been reported less frequently, mainly in case reports or small series, focusing on a single manifestation. Of note, the present study confirms the female predominance of symptomatic infection and the occurrence between age 20 and 40 years in most cases [4–7, 9].

A few studies in adults have described symptoms and clinical characteristics of HPV-B19 infection [2, 4–10, 17]. Like these earlier studies, we found joint and cutaneous manifestations to be common and had a favourable outcome in most cases, with good response to NSAIDs [2, 4–10]. However, in our patients, joint symptoms persisted 6 months after onset in 28% of patients versus 8 to 20% in previous series [2, 5, 6, 9]. In 2 of our patients, in whom rheumatoid factor was detected, RA was subsequently diagnosed. Although HPV-B19 infection preceding or mimicking RA has been reported [29], the presence of ACPA and joint destruction has not been described until now. Regarding cutaneous manifestations, our series is similar to previous series but highlights an association of Sweet’s syndrome and HPV-B19 infection, only reported twice before [14, 30]. Atypical extra-haematological manifestations such as renal injury, neurological complications or myocarditis associated with HPV-B19 have been less described in previous series and represented 12% of our patients. In our series, 52% patients presented manifestations other than joint or cutaneous features (i.e., renal, neurological, muscular, hepatic, pleuro-pericardial or splenic involvement), which highlights the need to seek these complications in patients with HPV-B19 infection. Of note, the present study adds a fifth case of Guillain-Barré syndrome related to HPV-B19 infection (patient 22) [31].

In the present study, most patients had immunological abnormalities (65.2%), which resolved in a few weeks. As for other viral infections, HPV-B19 infection can induce the production of various auto-Abs [26]. ANA and/or anti-dsDNA Abs were identified in more than 25% of patients in previous studies, versus 39 and 22%, respectively, in our series [9, 32]. Anti-phospholipid Abs were also frequently found, without clinical significance, in 14% of patients in one series, versus 36% in our study [9]. Low C4 level was previously reported in 32 to 44% of patients, which agrees with our results (39%) [4, 7, 9]. Thus, HPV-B19 infection may mimic both clinical and laboratory features of systemic lupus erythematosus (SLE), which justifies the need to screen for HPV-B19 infection when patients are referred for suspected SLE. Misdiagnosing systemic vasculitis is another difficulty: Hermann et al. found that 10% of patients with acute HPV-B19 infection (20% in the present series) had ANCA [33]. Moreover, cases of necrotizing vasculitis associated with HPV-B19 infection and cases of SLE triggered by HPV-B19 have been reported [23, 34].

HPV-B19 infection in adults is usually associated with a good outcome and full recovery. However, some patients experience severe manifestations and/or prolonged symptoms. In these patients, immunomodulatory and anti-inflammatory agents such as corticosteroids and IVIg are needed. As in our series, corticosteroids are empirically the first-line treatment to be chosen. With its immunomodulatory and anti-inflammatory but also anti-infective properties, IVIg has been proposed to treat extra-haematological manifestations associated with HPV-B19 infection. Indeed, IVIg is only validated in PRCA in immunocompromised patients. Indeed, in 93% patients reviewed by Crabol et al.*,* haemoglobin level was normalized at a mean of 1.7 ± 1.6 months after the first IVIg course [20]. To date, use of IVIg to treat manifestations related to HPV-B19 infection has been evaluated in only one prospective uncontrolled study in the setting of dilated cardiomyopathy and reported in retrospective case studies and case reports [13, 21–23, 35–43]. Although clinical improvement was reported in several cases, particularly for cardiac, vascular, and neurological involvement, relapses and/or sequelae frequently occurred. Moreover, discrepancies between clinical and virological responses were observed [13, 21–23, 35–43]. In the present study, 6 patients (24%) received IVIg. Half of them, especially those with neurological manifestations, showed clinical improvement. As expected, patient 22, with Guillain-Barré syndrome, had a favourable outcome. Of note, both patients with peripheral neuropathy (patients 20 and 21) also experienced clinical improvement.

The present study has several limitations. Its retrospective design explains the missing data, including, for example, serial measurement of viral load. The number of cases is limited because of the low incidence of extra-haematological manifestations of HPV-B19 infection. Also, another possible explanation is the potential bias in B19 testing for uncommon clinical presentations related to B19 infections. Indeed B19 testing would always be considered in adults presenting with arthralgia and/or rash, whereas it would never or rarely be considered in patients presenting with kidney or neurological manifestations. Another limitation in our study is the variety of manifestations which precludes any statistical analysis to identify prognostic factors or to assess treatment efficacy. Finally, when assessing corticosteroids or IVIg efficacy, progressive spontaneous improvement cannot be ruled out.

## Conclusions

In conclusion, our results confirm that HPV-B19 infection should be considered in the differential diagnosis assessment of a wide range of manifestations. Extra-haematological manifestations associated with HPV-B19 infection usually have a favourable outcome with supportive treatment. Furthermore, severe injury can occur, especially with neurological involvement: in these rare cases, IVIg treatment could be discussed, although its efficacy remains to be documented. Hence, more prospective studies are needed to define the place of IVIg in the management of extra-haematological manifestations associated with HPV-B19 infection [44].

## Abbreviations

Abs: Antibodies
ACPA: Anti-citrullinated Abs
ANA: Antinuclear Abs
ANCA: Anti-neutrophil cytoplasmic Abs
Anti-dsDNA: Anti-double stranded DNA
CRI: Club rhumatismes et inflammations
CRP: C-reactive protein
ESRD: End-stage renal disease
GN: Glomerulonephritis
HPV-B19: Human parvovirus B19
IVIg: Intravenous immunoglobulin
NSAIDs: Non-steroidal anti-inflammatory drugs
PCR: Polymerase chain reaction
PPGSS: Papulo-purpuric gloves-and-socks syndrome
PRCA: Pure red cell aplasia
RA: Rheumatoid arthritis
SLE: Systemic lupus erythematosus
SNFMI: Société nationale Française de médecine interne
